# Metabolic profiling in major depressive disorder with high psychological resilience: changes in amino acid and carbohydrate metabolism

**DOI:** 10.1186/s12888-026-07798-4

**Published:** 2026-01-17

**Authors:** Runnan Yang, Xi Chen, Guifeng Tan, Jingyi Yang, Jiayu Du, Jing Li, Wenjing Li, Huaibing Wang, Hongru Zhu, Minlan Yuan, Wei Zhang

**Affiliations:** 1 Mental Health Center and Psychiatric Laboratory, West China Hospital, Sichuan University, No. 37 Guo Xue Xiang, Chengdu, Sichuan 610041 China; 2 Department of Mechanical and Aerospace Engineering, The Hong Kong University of Science and Technology, Clear Water Bay, Kowloon, Hong Kong China; 3 Med-X Center for Informatics, Sichuan University, Chengdu, 610041 China; 4 West China Biomedical Big Data Center, West China Hospital, Sichuan University, Chengdu, 610041 China

**Keywords:** MDD, Metabolomics, Psychological resilience, Arginine, Erythronic acid, Oxidative stress

## Abstract

**Background:**

Psychological resilience varies among major depressive disorder (MDD) patients, with some exhibiting high resilience. This challenges the notion of resilience as purely protective and suggests biological heterogeneity. Both resilience and MDD have been linked to metabolic alterations, but their independent and interactive effects remain unclear. This study aims to investigate how resilience and MDD jointly affect metabolic profiles, with a focus on identifying key metabolic and pathway alterations in high-resilience MDD patients compared to healthy controls, and exploring their potential for diagnostic biomarkers.

**Methods:**

Targeted serum metabolomics using UPLC-MS/MS was conducted in MDD patients and healthy controls. Resilience was assessed via the Ego Resiliency Scale (ERS). Interaction effect analysis examined the main and interactive influences of resilience and MDD. Key metabolites in high-resilience MDD were identified by OPLS-DA and pathway enrichment. A logistic regression model with cross-validation assessed diagnostic accuracy in training and test sets.

**Results:**

A total of 271 participants were enrolled, and about one-third of MDD patients exhibited high resilience. The MDD×resilience interaction was not significant, whereas MDD showed a significant main effect on metabolite levels. Five key metabolites were identified in high-resilience individuals, with arginine, methionine, and kynurenine downregulated and threonic and erythronic acids upregulated in the high-resilience MDD group. The diagnostic model achieved an area under the curve (AUC) of 0.811 in the test set.

**Conclusions:**

MDD status—rather than psychologically resilience—was the primary driver of serum metabolic variation. In high-resilience individuals, alterations converged on amino acid pathways (driven by lower arginine, methionine, kynurenine) and pentose-glucuronate axis (with higher threonic and erythronic acids), the latter is potentially linked to redox imbalance. These features may provide novel insights into depression-related metabolic dysregulation.

**Clinical trial number:**

Not applicable.

**Supplementary Information:**

The online version contains supplementary material available at 10.1186/s12888-026-07798-4.

## Introduction

Approximately 300 million people around the world suffer from depressive disorders [1]. In 2019, depression accounted for 1.85% of global disability-adjusted life-years (DALYs), with major depressive disorder (MDD) contributing 1.47% and dysthymia accounting for 0.38% [2]. As a chronic and recurrent mental illness, MDD is more severe than dysthymia and is characterized by persistent low mood, lack of interest, reduced energy, and even suicide, which severely impairs an individual’s daily life and social functions [3, 4]. According to the World Health Organization, MDD is predicted to be the leading cause of global disease burden by 2030 [5, 6]. Notably, MDD can be regarded as one of the stress-related psychiatric disorders [7]. Stress exposure—such as childhood maltreatment and recent stressful life events—as well as dysregulated responses, including passive coping strategies and overactivation of physiological stress response systems, could trigger this disease [8–10]. For instance, after the COVID-19 pandemic in 2020, the global prevalence of MDD increased by 27.6%, with a similar trend observed in China [11–13].

However, not everyone exposed to traumatic stress develops mood distress. Individuals who are resilient to such effects exhibit psychological resilience [14, 15], defined as the ability to actively engage in psychological or physiological adaptations in response to stress or threat, thereby facilitating recovery of well-being and fostering growth under adverse conditions [16, 17]. Research has shown that individual variations in resilience levels play a modulatory role in stress-related psychiatric disorders [18]. Typically, resilience serves as a protective factor against depression and anxiety [19, 20]. MDD patients often display diminished psychological resilience, and lower resilience is associated with increased severity and recurrence of the disorder [21–23]. Nevertheless, empirical studies indicate that a subset of depressed individuals shows high resilience levels comparable to healthy populations [24, 25]. Psychological resilience is not invariably positively correlated with mental health levels; indeed, individuals with high resilience may concurrently experience mental health problems [26]. A recent longitudinal cohort study on depressive symptom trajectories revealed that resilient individuals consistently exhibit lower depressive symptomatology levels during follow-up [27]. The heterogeneity of psychological resilience in depression may reflect distinct biological underpinnings. Characterizing biomarkers and pathways in high-resilience MDD individuals can deepen understanding of depression mechanisms and inform personalized, resilience-enhancing strategies.

Emerging evidence suggests that metabolic alterations are linked to the pathophysiology of stress-related psychiatric disorders, with metabolic abnormalities contributing to behavioral changes [28, 29]. Previous studies have indicated that peripheral plasma metabolomics is altered in MDD, involving impaired mitochondrial energy metabolism [30, 31] and disruptions in neuronal integrity and transmission [32]. These biomarkers may have potential applications in diagnosing, treating, and preventing MDD [33, 34]. Interestingly, recent animal studies have revealed that metabolic changes in the blood or brain of rats are associated with resilience to stress exposure [35–37]. In human studies, several lines of research have found distinct metabolites linked to resilience in healthy subjects [38] and depressive adolescents [39]. However, biological studies on psychological resilience, specifically within MDD populations, remain scarce [40]. Moreover, existing studies mainly compare patients and controls or high- and low-resilience groups, overlooking potential interaction effects between resilience level and disease status, particularly comparisons between MDD and healthy individuals within the same resilience strata.

This exploratory study adopted a multi-group comparative framework to examine metabolite differences across resilience levels in MDD patients and healthy controls. Particular focus was placed on high-resilience individuals with MDD, who may represent a biologically distinct subgroup with unique metabolic alterations. Identifying their distinct metabolic profiles could provide insights into the metabolic disruptions associated with high-resilience MDD, offering potential biomarkers for precision stratification of depression. To this end, we also developed a diagnostic model to detect MDD among high-resilience individuals based on key metabolites.

## Methods

### Participant recruitment

All MDD patients were screened and recruited from the outpatient psychiatric clinics at the Mental Health Center of West China Hospital, Sichuan University, from April 2021 to August 2024. Written informed consent was obtained from all participants prior to the clinical assessments and sample collection. Inclusion criteria were as follows: (1) Meeting the diagnosis criteria for major depression according to the Diagnostic and Statistical Manual of Mental Disorders 5th edition (DSM-5), as confirmed by the Mini International Neuropsychiatric Interview (M.I.N.I.) administered by two board-certified psychiatrists; diagnostic disagreements were resolved by consensus (first-onset or recurrent episodes); (2) A baseline score of ≥ 14 on the Hamilton Depression Rating Scale-17 items (HAMD-17) [41]; (3) Aged 18–65 years; (4) No medication or discontinued psychiatric medication for over 2 weeks (exceptions apply to sedative drugs); (5) A minimum of a primary school education was required to comprehend the study content. Exclusion criteria were as follows: (1) Schizophrenia, substance abuse, mental retardation, neurological anorexia/bulimia, and personality disorder, based on the M.I.N.I.; (2) Severe physical diseases, epilepsy, seizures, severe cognitive impairment or central nervous system diseases; (3) Inability to obtain Ego-Resiliency Scale (ERS) information; [4] Refusal to sign the informed consent form or inability to complete evaluation questionnaires. We also recruited healthy controls at the same time, who were free of any psychiatric diagnoses as confirmed by M.I.N.I. The study adhered to the principles of the Declaration of Helsinki and received approval from the Ethics Committee of West China Hospital (No. 20201054).

### Clinical assessments

Psychological resilience was measured using the ERS, initially developed by Block and Kremen [42], with the Chinese version of the scale employed in this study [43]. It utilizes a 1-to-4 point Likert scale ranging from 1 (does not apply at all) to 4 (applies very strongly), comprising 14 items. Higher scores are indicative of higher resilience. In this study, Cronbach’s alpha was found to be 0.86. The median score on the scale for all participants in this study was 37, with a standard deviation of 7.6. Therefore, we used the median split [37] to classify participants into high (ERS score ≥ 37) and low (ERS score < 37) resiliency groups. This median-based grouping has been widely adopted in resilience research when no universally accepted external cut-off is established [44, 45]. Participants were further divided into four groups: high-resilience MDD patients (HRD), high-resilience healthy controls (HRC), low-resilience MDD patients (LRD), and low-resilience healthy controls (LRC).

The severity of depressive and anxiety symptomatology was evaluated by HAMD-17 and the Hamilton Rating Scale for Anxiety (HAMA-14) [46]; higher scores indicated a more significant presence of depressive and anxiety symptoms. The Childhood Trauma Questionnaire-Short Form (CTQSF) [47] scale was used to measure individuals with histories of abuse and neglect during childhood, which includes 28 items ranging from 1 (never been through) to 4 (always been through). The scale’s total score ranges from 25 to 125, with higher scores indicating more severe maltreatment histories. Other measurements include the body measure index (BMI), education level, employment status, and marital status. All patients and healthy controls completed the above clinical assessments, along with demographic measures.

### Blood sampling

Blood samples (2 ml per sample) of venous blood from participants were collected at the time of initial diagnosis and allowed to rest for 30 min before centrifugation. The serum was separated from the whole blood samples after centrifugation (3000 rpm at 4 °C for 15 min) and immediately stored at -80 °C until laboratory testing.

### Metabolites analysis

Targeted metabolomics was performed using the Q300 kit (Metabo-Profile, Shanghai, China). This automated high-throughput metabolite array technology can rapidly and quantitatively determine 300 plus metabolites including fatty acids, amino acids, organic acids, carbohydrates, and bile acids (10.1021/acs.analchem.0c04686). An ultraperformance liquid chromatography coupled to tandem mass spectrometry (UPLc.MS/MS) system (ACQUITY UPLC-Xevo TQ-s, Waters Corp., Milford, MA, USA) was used to quantitate all targeted metabolites in this study (Metabo-Profile Biotechnology [Shanghai] Co. Ltd). The optimized instrument settings are briefly described as follows. For HPLC, column: ACQUITY HPLCBEH C18 17 × 10–6 m VanGuard precolumn (2.1 × 5 mm) and ACQUITY HPLC BEH C181.7 × 10–6 m analytical column (2.1 × 100 mm), column temp.: 40 °C, sample manager temp.: 10 °C, mobile phases: A = water with 0.1% formic acid; and B = acetonitrile/PA (70 − 30), gradient conditions: 0–1 min (5% B), 1–11 min (5–78% B), 11–13.5 min (78–95% B), 13.5–14 min (95–100% B), 14–16 min (100% B), 16–16.1 min (100-5%B), 16.1–18 min (5% B), flow rate: 0.40 mL min-1, and injection vol: 5.0 uL. For the mass spectrometer, capillary:1.5 (ESl+), 2.0 (ES1-) Kv, source temp.: 150 °C, desolvation temp.: 550 °C, and desolvation gas flow: 1000 L h-1. For data processing, the raw data files generated by UPLC-MS/MS were processed using TMBQ software (v1.0, Human Metabolomics Institute, Shenzhen, Guangdong, China) for peak integration, calibration, and quantification of each metabolite. The self-developed platform iMAP (v1.0, Metabo-Profile, Shanghai, China) was used to calculate the concentration of each analyte in the samples. Metabolite levels were log2-transformed before analysis.

Metabolomic data were analyzed using the limma package in R (version 4.3.1). We specified per-metabolite linear models including MDD status, psychological resilience group, and their interaction (MDD × Resilience), adjusting for age and gender. Potential collinearity among predictors was assessed using the Generalized Variance Inflation Factor (GVIF) and present GVIF^1/(2⋅*Df*)^. Sensitivity analyses additionally adjusted for symptom severity (HAMD-17, HAMA-14) and childhood adversity (CTQSF) to evaluate robustness. Model fitting was performed using the lmFit function, followed by empirical Bayes moderation with the eBayes function. The significance of the main effects and interaction effects was assessed using the topTable function. P-values were adjusted for multiple comparisons using the Benjamini–Hochberg false discovery rate (FDR) method. In the primary analysis of the main effect of MDD, an adjusted p-value < 0.05 and |log fold change (logFC)| > 0.1 were applied to identify significantly different metabolites. This approach allowed us to detect metabolites with subtle yet potentially biologically meaningful differences between MDD and healthy controls. For the subgroup analysis focusing on high-resilience individuals, a more stringent threshold of |logFC| > 0.5 combined with variable importance in projection (VIP) > 1.5, based on orthogonal partial least squares discriminant analysis (OPLS-DA), was applied to select key metabolites with larger effect sizes and greater biological relevance. Model validation was performed using 200-times permutation tests, confirming the robustness and reliability of the OPLS-DA model. Volcano plots were created to visualize the changes in fold change and their significance. Functional enrichment analysis was conducted through MetaboAnalyst 5.0 using the Kyoto Encyclopedia of Genes and Genomes (KEGG) database. Pathways were considered significantly enriched at *p* < 0.05.

### Construction of a diagnostic model

The high resilience group participants were randomly divided into a training set (70%) and a testing set (30%). Key differential metabolites identified within this subgroup were used to construct a logistic regression model in the training set. Ten-fold cross-validation was performed within the training set to evaluate model stability and prevent overfitting. The diagnostic performance of the model was then validated in the testing set. Receiver operating characteristic (ROC) curve analysis was performed to evaluate the model’s performance, with the area under the curve (AUC) as the primary metric. We interpreted AUC using standard conventions (0.7–0.8 acceptable, 0.8–0.9 good, > 0.9 excellent) [48, 49].

### Statistical analysis

All data in this study were analyzed using iMAP and R 4.3.1 software. Continuous variables were presented as means ± standard deviation (SD), and categorical variables were presented as quantities and percentages (%). The sociodemographic and clinical variables between HRD, LRD, HRC, and LRC were compared using two independent-sample Mann-Whitney U test and Chi-square test, respectively. Partial Spearman correlation analysis with control for covariates was conducted to assess associations between clinical variables and interindividual metabolite variations in highly resilient individuals. Bonferroni correction was used for multiple comparisons of demographic variables, while the FDR method was used for metabolomic data. Statistical significance was set at a threshold of *p* < 0.05 (two-tailed).

## Results

### Participants demographics

Plasma metabolite data were obtained from 271 participants. Demographic and clinical characteristics are summarized in Table 1. Notably, around one-third of individuals with MDD demonstrated high resilience in our sample (58/174). Significant group differences were found in gender distribution (*p* < 0.001), age (*p* = 0.010), HAMD-17 (*p* < 0.001), HAMA-14 (*p* < 0.001), resilience levels (*p* < 0.001), and CTQSF scores (*p* < 0.001) among four groups. Most participants were young to middle-aged adults, with the majority holding a bachelor’s degree or higher. Males comprised over 50% of the HRC group. The HRD group had the highest mean age (38.4 ± 16.2 years). BMI was generally within the normal range, though the LRC group showed a trend toward overweight without significant group differences. MDD patients had significantly higher depression and anxiety scores than healthy controls, regardless of resilience, with the LRD group showing the highest symptom severity. The LRD group also reported the highest childhood trauma scores (49.0 ± 14.6). Among healthy controls, childhood trauma scores did not significantly differ by resilience level.

**Table 1:** Participants characteristics and clinical assessments

	MDD		HC		Pt	*P*. adj			
	HR (*n*=58)	LR (*n*=116)	HR (*n*=84)	LR (*n*=13)		HRD vs. LRD	HRC vs. LRC	HRD vs. HRC	LRD vs. LRC
**Gender**,** n (%)**					<0.001 ^b*^	0.181 ^a^	0.181 ^a^	0.042^a*^	0.951 ^a^
male	17 (29.3)	21 (18.1)	44 (52.4)	3 (23.1)					
female	41 (70.7)	95 (81.9)	40 (47.6)	10 (76.9)					
**Age**,** years**,** mean (SD)**	38.4 (16.2)	31.5 (11.6)	33.8 (11.3)	33.0 (9.5)	0.010 ^c*^	0.054 ^c^	0.937 ^c^	0.409 ^c^	0.695 ^c^
**BMI**,** kg/m**^**2**^, **mean (SD)**	22.3 (4.3)	21.8 (4.8)	22.6 (3.7)	25.5 (10.2)	0.064 ^c^	0.592 ^c^	0.920 ^c^	0.592 ^c^	0.592 ^c^
**Education**,** n (%)**					0.489 ^b^	1 ^a^	0.808 ^a^	0.808 ^a^	1 ^a^
Below Bachelor’s degree	14 (24.1)	28 (24.1)	14 (16.7)	4 (30.8)					
Bachelor’s degree or higher	44 (75.9)	88 (75.9)	70 (83.3)	9 (69.2)					
**Marital status**,** n (%)**					0.545 ^b^	0.659 ^a^	0.659 ^a^	0.659 ^a^	0.659 ^a^
Married	29 (50.0)	48 (41.4)	35 (41.7)	4 (30.8)					
other	29 (50.0)	68 (58.6)	49 (58.3)	9 (69.2)					
**employment status**,** n (%)**					0.406 ^b^	1 ^a^	1 ^a^	1 ^a^	1 ^a^
Full-time employment	35 (60.3)	60 (51.7)	53 (63.1)	7 (53.8)					
Unemployment	23 (39.7)	56 (48.3)	31 (36.9)	6 (46.2)					
**HAMD-17**,** mean (SD)**	17.4 (4.3)	18.0 (5.3)	2.2 (2.0)	2.6 (2.1)	<0.001 ^c*^	0.800 ^c^	0.562 ^c^	<0.001 ^c*^	<0.001 ^c*^
**HAMA-14**,** mean (SD)**	19.3 (7.4)	21.8 (7.8)	1.9 (3.4)	1.5 (1.5)	<0.001 ^c*^	0.118 ^c^	0.655 ^c^	<0.001 ^c*^	<0.001 ^c*^
**ERS**,** mean (SD)**	41.4 (3.5)	30.0 (4.3)	44.0 (4.8)	33.4 (2.2)	<0.001 ^c*^	<0.001 ^c*^	<0.001 ^c*^	<0.001 ^c*^	0.004 ^c*^
**CTQSF**,** mean (SD)**	42.2 (14.1)	49.0 (14.6)	33.5 (7.0)	40.0 (13.3)	<0.001 ^c*^	0.001 ^c*^	0.076 ^c^	<0.001 ^c*^	0.010 ^c*^

BMI, body mass index; MDD major depression patients; HC, healthy controls; HR, high resilience; LR, low resilience; HRD, high resilience major depressive disorder patients; LRD, low resilience major depressive disorder patients; HRC, high resilience healthy controls; LRC, low resilience healthy controls; HAMD-17, the Hamilton Depression Rating Scale-17 items; HAMA-14, the Hamilton Rating Scale for Anxiety Scale; ERS, the Ego-Resiliency Scale; CTQSF, the Childhood Trauma Questionnaire-Short Form; P. adj, p-value was Bonferroni corrected

^a^: Chi-square test; ^b^: Fisher’s exact test; ^c^: Mann-Whitney U test; ^⁎^: Pt or P. adj<0.05 is statistically significant

### Effect of MDD status, psychological resilience, and their interaction effects on metabolomic profiles

In this study, a total of 186 metabolites were detected by targeted metabolomics. The initial interaction analysis suggested a significant interaction between psychological resilience and MDD status on metabolite levels, with methylglutaric acid showing a significant interaction before adjusting for covariates (adjusted p-value = 0.018). After adjusting for age and gender, the interaction effect was no longer significant (e.g., methylglutaric acid adjusted p-value = 0.053, Table S1 in Supplementary Material 1). We then conducted sensitivity models that additionally adjusted for HAMD-17, HAMA-14 and CTQSF. Under these models, no metabolite showed an MDD×resilience interaction that survived FDR correction (0/186); for methylglutaric acid the signal further attenuated (adjusted p-value = 0.070). A stratified permutation test (within LR/HR strata; 2,000 shuffles) indicated the observed minimum FDR (adjusted p-value = 0.053) was not extreme (empirical p-value = 0.25), supporting the robustness of the null interaction finding. As BMI showed no between-group differences (Table S2), it was not retained, suggesting that BMI did not confound the relationship between psychological resilience and MDD status. No concerning collinearity was detected among predictors (GVIF^1/(2⋅D*f*)^ range 1.02–1.06).

**Fig. 1:**
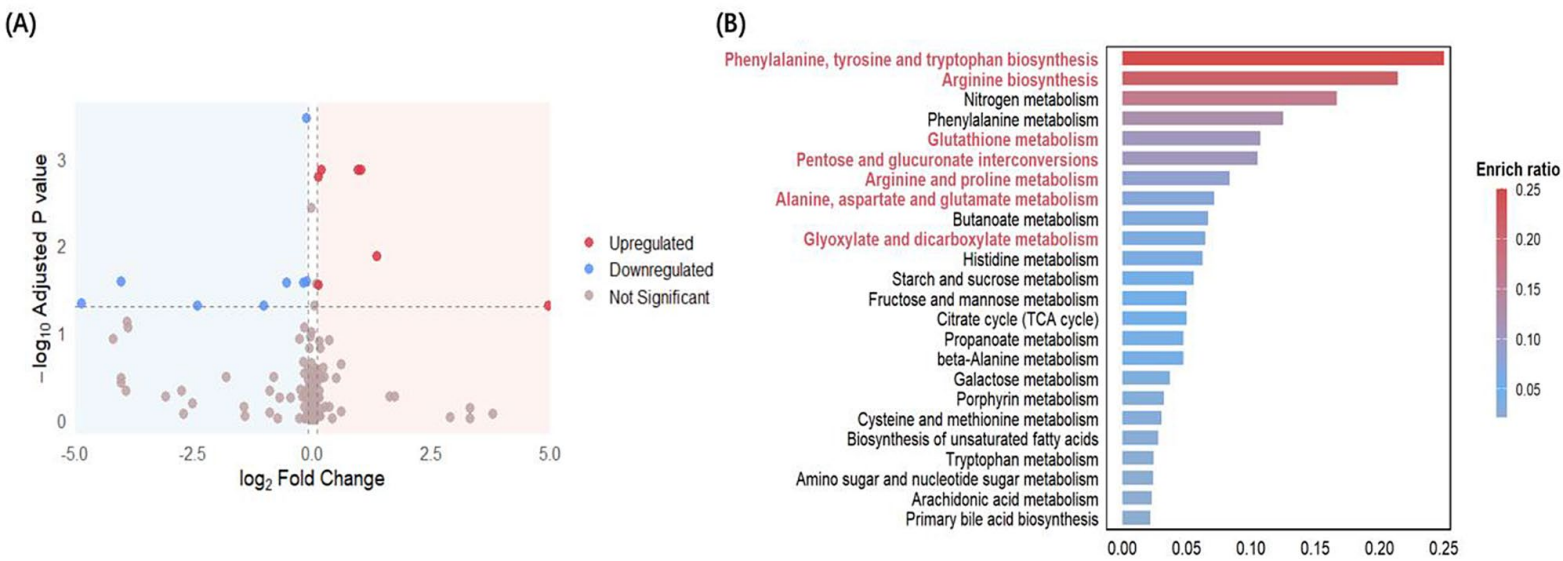
Metabolic alterations and pathway enrichment associated with MDD: main effect analysis. (**A**) Volcano plot showing differential metabolites between MDD patients and healthy controls. Red dots represent significantly upregulated metabolites, and blue dots represent significantly downregulated metabolites (adjusted p-value < 0.05, |logFC| > 0.1). Gray dots indicate non-significant metabolites. (**B**) KEGG pathway enrichment analysis of the differential metabolites. Enriched metabolic pathways are shown, with color representing the enrichment ratio. Pathways marked in red represent those with statistically significant enrichment (*p* < 0.05)

As the MDD×resilience interaction did not survive adjustment, we proceeded to estimate main effects in the age/gender-adjusted primary model. This analysis revealed a significant effect of MDD but not psychological resilience. A relatively lenient significance threshold was applied to capture a comprehensive profile of metabolic alterations associated with MDD (adjusted p-value < 0.05 and |logFC| > 0.1). In total, 20 significantly altered metabolites were identified in patients with MDD compared to healthy controls, regardless of resilience levels (Fig. 1A). Among them, the five most upregulated metabolites were ornithine, pyroglutamic acid, glutamic acid, threonic acid, and erythronic acid; the most downregulated were arginine, citric acid, hydroxypropionic acid, fructose, and methionine (Table S3). Pathways related to amino acid metabolism, including phenylalanine, tyrosine, tryptophan, and arginine biosynthesis, were significantly enriched in MDD patients (Fig. 1B, Figure S1). Notably, within this pathway, tryptophan showed no significance after FDR adjustment (logFC = -3.882, *p* = 0.013, adjusted p-value = 0.089). It, therefore, was not included in Table S3; given the well-established role of the kynurenine-tryptophan pathway in neuroinflammation and neurotransmitter synthesis of involvement in MDD, we observe the significant difference of the ratio of kynurenine (KYN) to tryptophan (TRP) in HC and MDD patients (mean ratio: 0.065, 0.059, respectively, *p* = 0.010), these findings still worth further discussion.

It is remarkably, however, apart from amino acid metabolism, the pentose and glucuronate Interconversion pathway was the most significantly enriched non–amino acid metabolic pathway (Fig. 1B), which is related to carbohydrate metabolism. Ribulose (logFC = 0.187, adjusted p-value = 0.001) and xylulose (logFC = 0.113, adjusted p-value = 0.028), two key metabolites in this pathway, tended to be higher in MDD patients than in healthy controls (Table S3).

### Distinct metabolic alterations in high resilience individuals with MDD

We then examined disease-related differences within a uniform resilience context by contrasting HRD and HRC. Differential metabolites were identified with limma (adjusted p-value < 0.05 and |logFC | > 0.5) and further screened by OPLS-DA (VIP > 1.5). Results for the low resilience stratum are provided for completeness in Table S4 but are not interpreted due to severe imbalance (116 MDD vs. 13 HC).

**Fig. 2:**
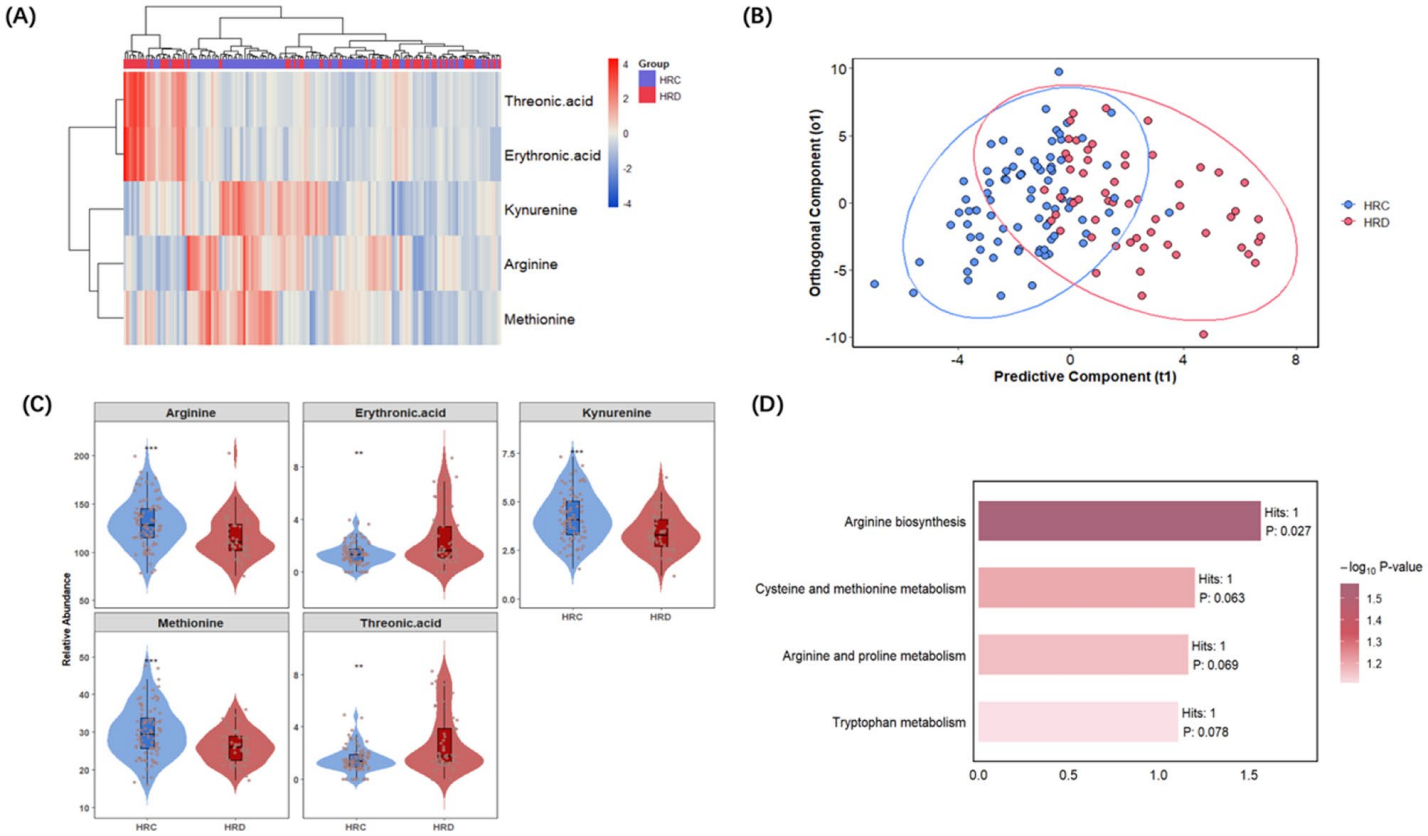
Metabolomic analysis of differential metabolites between MDD patients and healthy controls with high resilience levels. (**A**) Heatmap of five significant differential metabolites. Each row represents a metabolite, and each column represents an individual sample. Red indicates higher expression levels, while blue indicates lower levels. Rows and columns are clustered by hierarchical clustering (Euclidean distance; Ward.D2 linkage) on row-wise z-scored values. (**B**) OPLS-DA score plot showing metabolic discrimination between MDD patients (HRD, light coral) and healthy controls (HRC, light blue). Each point represents a sample, and the ellipses indicate each group’s 95% confidence intervals. (**C**) Violin & Boxplot of five significant differential metabolites level. Red represents MDD patients, and blue represents healthy controls. Statistical significance was assessed using the Mann-Whitney U test. ^⁎^:*p*<0.05; ^⁎⁎^:*p* < 0.01; ^***^: *p* < 0.001. (**D**) KEGG pathway enrichment bar plot. The bar length and color both represent the statistical significance of enrichment (− log₁₀ P-value), with darker colors indicating higher significance. The number of differential metabolites (Hits) and corresponding p-values are labeled on the bars

The model showed satisfactory fitness (R2Y = 0.486) and robustness, with validation by permutation testing (pR2Y = 0.025, pQ2 = 0.005, Figure S2). The results revealed distinct expression profiles of these metabolites between HRD and HRC (Fig. 2A and B). As shown in Table 2, five key differential metabolites were identified. Specifically, the arginine, methionine, and kynurenine levels markedly decreased in the HRD group compared to the HRC group. A similar trend was observed in the ratio of KYN to TRP (logFC = -4.115, *p* = 0.029, adjusted p-value = 0.223, VIP score = 1.581), with a decrease in HRD compared to HRC (mean ratio: 0.060, 0.066, respectively, *p* = 0.070). At the same time, threonic acid and erythronic acid exhibited higher abundance in HRD relative to HRC individuals (Fig. 2C). These findings suggest that even among individuals with high psychological resilience, MDD is associated with marked disruptions in key metabolic pathways.

KEGG pathway enrichment analysis was performed further to explore the potential biological significance of these differential metabolites. The results indicated that these metabolites were primarily enriched in arginine biosynthesis (*p* = 0.027), cysteine and methionine metabolism (*p* = 0.063), arginine and proline metabolism (*p* = 0.069), and tryptophan metabolism (*p* = 0.078) pathways (Fig. 2D, Figure S3). These pathways mainly involve amino acid metabolism, neurotransmitter synthesis, and oxidative stress regulation.

**Table 2:** Key differential metabolites between HRC and HRD groups

Metabolite	logFC	*P*	*P*. adj	VIP scores	Direction (HRD vs. HRC)
Arginine	-15.736	<0.001	0.017	2.184	↓
Methionine	-3.722	<0.001	0.025	1.710	↓
Threonic acid	1.217	<0.001	0.009	2.936	↑
Erythronic acid	1.073	<0.001	0.011	3.007	↑
Kynurenine	-0.604	0.002	0.042	1.762	↓

VIP, variable importance in projection; HRD, high resilience major depressive disorder patients; HRC, high resilience healthy controls; P. adj, p-value was Bonferroni corrected

Overall, the HRD–HRC pattern mirrors the full cohort main effect, focusing on amino acid metabolism and the pentose and glucuronate Interconversion pathways, with the five key metabolites mapping to these axes.

### Potential biomarkers for identifying MDD among individuals with high resilience levels

Based on the five identified key differential metabolites, a logistic regression model was developed in the training set (35 MDD patients vs. 64 healthy controls). Ten-fold cross-validation was performed to internally validate the model, resulting in an average AUC of 0.761.

**Fig. 3:**
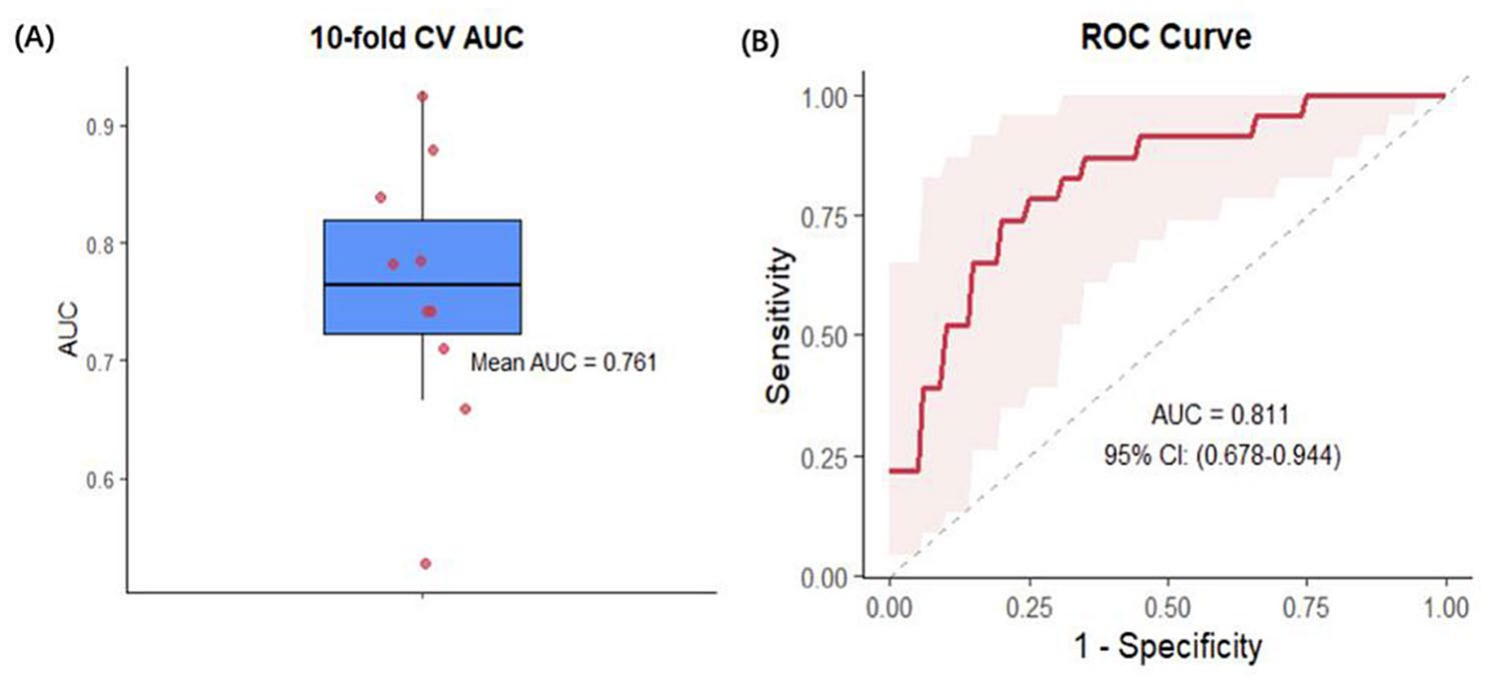
Validation of logistic regression model based on five key differential metabolites. (**A**)Cross-validation performance: A ten-fold cross-validation was conducted using the training set (35 MDD patients vs. 64 healthy controls). The boxplot shows the distribution of AUC for each fold, with a mean AUC of 0.761, indicating moderate discriminative ability. (**B**) External validation performance: The model was externally validated in the testing set (23 MDD patients vs. 20 healthy controls). The ROC curve demonstrates a higher AUC of 0.811, with a sensitivity of 60.9% and specificity of 85.0%, showing promising diagnostic performance for identifying MDD among individuals with high psychological resilience. The shaded area represents the 95% confidence interval for the ROC curve

External validation in the testing set (23 MDD patients vs. 20 healthy controls) demonstrated a higher AUC of 0.811 (0.678–0.944), with a sensitivity of 60.9% and a specificity of 85.0% (Fig. 3), indicating promising diagnostic performance for identifying MDD among individuals with high psychological resilience.

### Correlation between key differential metabolites and clinical variables in individuals with high resilience

**Fig. 4:**
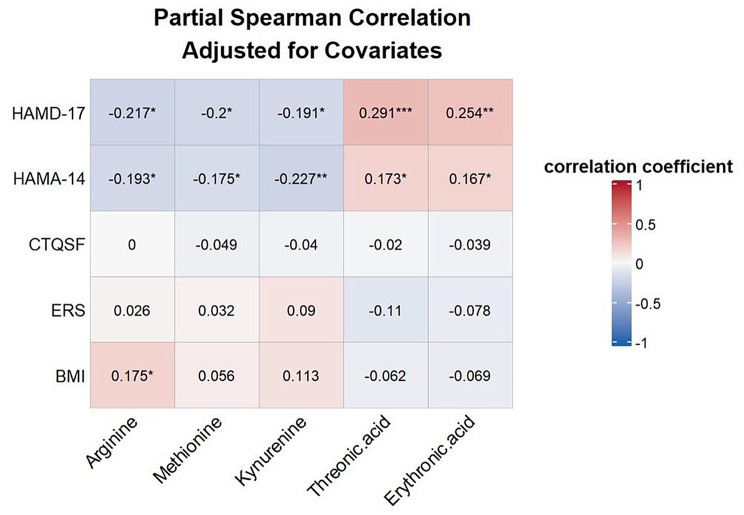
Partial Spearman correlation analysis between key differential metabolites and clinical variables in individuals with high resilience. The heatmap shows the partial Spearman correlation coefficients between five key differential metabolites and clinical variables in the high resilience group, adjusted for age and gender. The color gradient represents the strength and direction of the correlations, with red indicating positive correlations and blue indicating negative correlations. The numeric values in each cell represent the correlation coefficients. HAMD-17, the Hamilton Depression Rating Scale-17 items; HAMA-14, the Hamilton Rating Scale for Anxiety Scale; CTQSF, the Childhood Trauma Questionnaire-Short Form; ERS, the Ego-Resiliency Scale; BMI, the body measure index. *: FDR-adjusted p-value < 0.05

Partial Spearman correlation analysis revealed that several key differential metabolites were closely associated with depression and anxiety severity in the high resilience group (Fig. 4). Notably, higher levels of threonic acid (*r* = 0.291, adjusted p-value < 0.05) and erythronic acid (*r* = 0.254, adjusted p-value < 0.05) were positively correlated with increased depression severity, remaining significant after FDR correction. Conversely, lower levels of arginine, methionine, and kynurenine tended to be associated with reduced depressive symptoms, though these correlations did not remain significant after FDR correction. No significant correlations were found between the key metabolites and BMI, CTQSF, or ERS scores. The correlation heatmap for all variables is presented in Supplementary Fig. 4.

## Discussion

Metabolic alterations associated with depression vulnerability and resilience have been previously reported, with a series of differential metabolites identified in the plasma and brain tissues of animal models involving multiple metabolic pathways [37, 50, 51]. Understanding the impact of depressive states and psychological resilience on metabolic changes is crucial. In the present study, we demonstrate for the first time that MDD status is the primary factor driving metabolic alterations independent of resilience. We also identify five key differential metabolites between high-resilience MDD patients and healthy controls, implicating disruptions in amino acid and glucose metabolism pathways. This differential metabolite panel holds potential as a diagnostic biomarker for MDD in high-resilience populations.

Firstly, our study revealed a fascinating finding: over 40% of individuals with high resilience were diagnosed with MDD, while 13% of healthy individuals exhibited low resilience. This suggests that psychological resilience and mental health outcomes are complex and not necessarily correlated in a straightforward manner. This phenomenon is also supported by previous research [26, 52]. Furthermore, it may highlight that the protective effect of resilience against mood disorders is limited, with other factors playing a more prominent role in the onset of MDD, surpassing the influence of resilience. Our study provides preliminary evidence suggesting that metabolic dysfunction may be one such critical factor: even in individuals with high psychological resilience, altered metabolic states may override protective psychological mechanisms, ultimately contributing to the onset of depression.

The main effect analysis confirmed that disease status has the most significant impact on metabolic changes in MDD patients. The metabolites identified in the high-resilience subgroup analysis showed significant changes in line with the overall MDD effect. In particular, arginine was a distinctive metabolite, showing the most significant decrease in high-resilience MDD individuals. At the same time, ornithine exhibited the most prominent increase in the overall MDD group. Arginine is catabolized into ornithine via the urea cycle and involved in the synthesis of nitric oxide (NO), which plays a role in alleviating vascular tension and regulating stress symptoms [53, 54]. These findings suggest that the decreased bioavailability of arginine may contribute to the development of MDD, which is consistent with the most existing research [55, 56]. Additionally, we found that methionine levels were significantly decreased in both the high-resilience MDD subgroup and the overall MDD group. The downregulation of methionine disrupts the synthesis of S-adenosylmethionine (SAMe). This critical methyl donor has been reported to be reduced in the cerebrospinal fluid of MDD patients [57], thereby limiting the synthesis of brain neurotransmitters and compromising neuronal membrane stability [58, 59]. Growing evidence suggests that methionine is a key differential metabolite in depression, with significant downregulation observed in both adolescent and adult MDD patients [60, 61]. Besides, the kynurenine-tryptophan metabolic pathway is known to be implicated in depression. In line with results from a large sample meta-analysis [28], we observed that both kynurenine levels and the KYN/TRP ratio were significantly reduced in MDD patients, irrespective of resilience levels. Tryptophan serves as a critical precursor for serotonin, and it is metabolized into kynurenine through the action of tryptophan 2,3-dioxygenase. As a neuroprotective factor, a decrease in kynurenine levels may indicate increased neurotoxic burdens during the course of depression [62, 63]. However, the current results are inconsistent. Some studies have reported a decrease in kynurenine and tryptophan levels in treatment-naïve depressed patients [64, 65], whereas these levels tend to increase with regular antidepressant use [66]. On the other hand, some studies suggest that both tryptophan and kynurenine levels steadily decline in MDD patients, regardless of medication status [28]. Future research will require more subgroup analyses based on drug exposure to provide additional evidence.

**Fig. 5:**
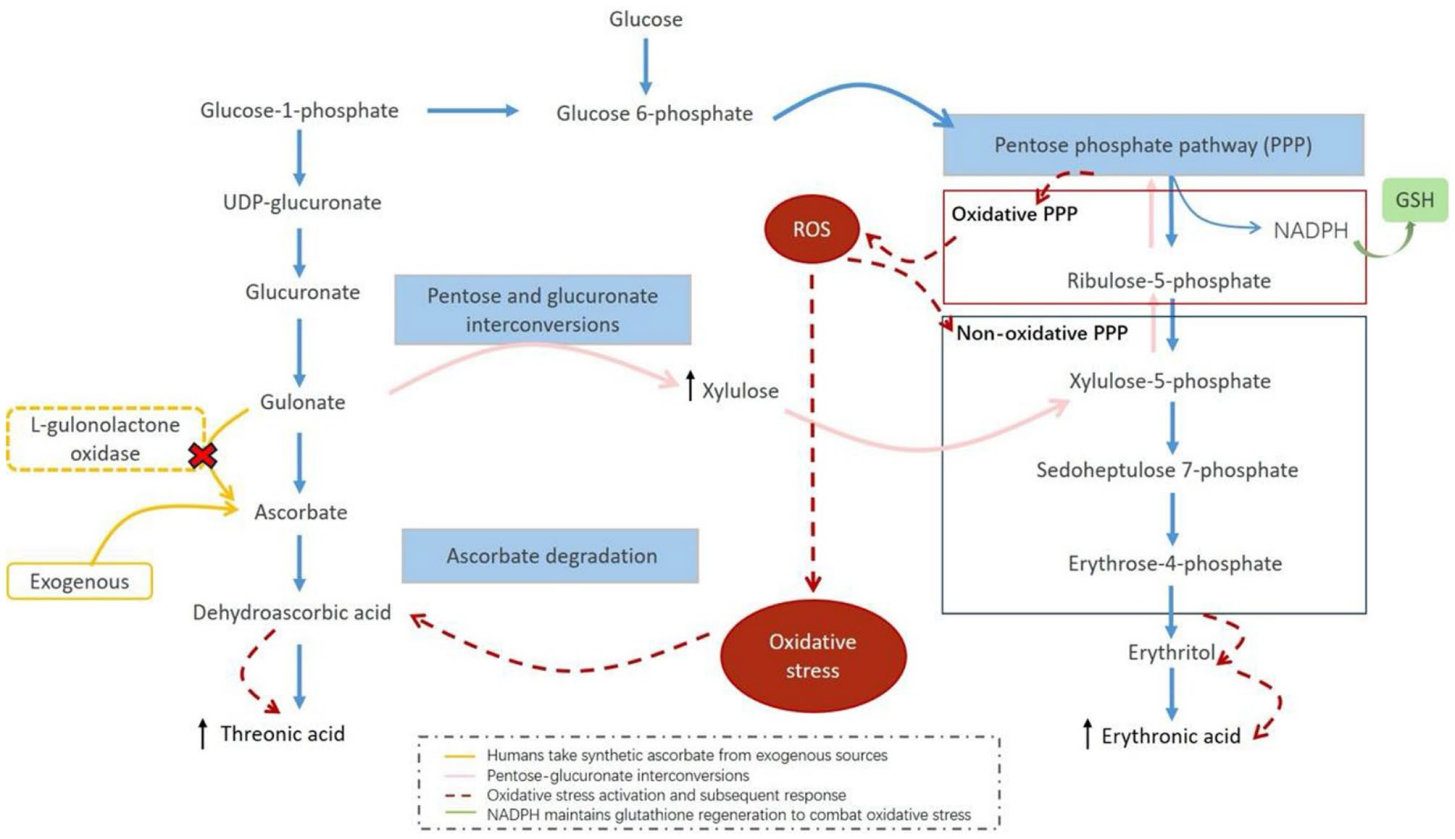
Metabolic origins of threonic acid and erythronic acid in depression and their relation to oxidative stress. Threonic acid is a downstream product of ascorbate (vitamin C) metabolism, which relies entirely on dietary intake due to the absence of L-gulonolactone oxidase in humans. Ascorbate plays a crucial role in antioxidative defense, and its degradation is influenced by redox balance. When reactive ROS levels increase, ascorbate is rapidly oxidized to dehydroascorbic acid and subsequently degraded to threonic acid, reflecting excessive vitamin C turnover. Erythronic acid is derived from erythritol metabolism via the PPP. Perturbations in PPP, potentially driven by an increased demand for NADPH in redox regulation, may lead to metabolic imbalances that compromise antioxidant capacity. The upregulation of glucuronate metabolism, which is involved in detoxification and oxidative stress adaptation, further suggests a shift in redox homeostasis. Together, these metabolic changes highlight the intricate interplay between glucose metabolism, oxidative stress, and biochemical alterations observed in depression. ROS, reactive oxygen species; PPP, pentose phosphate pathway; GSH, glutathione

Intriguingly, beyond the well-established perturbations in amino acid metabolism observed in the peripheral blood of depressed patients, our study highlights dysregulated carbohydrate metabolism, particularly in pentose and glucuronate interconversions, the pentose phosphate pathway (PPP), and ascorbate metabolism. Specifically, threonic acid and erythronic acid were significantly elevated in both the overall MDD cohort and the HRD subgroup, correlating positively with depressive and anxiety symptoms in high-resilience individuals. Threonic acid, a downstream ascorbate metabolite, accumulates due to enhanced ascorbate degradation, reflecting impaired reactive oxygen species (ROS) clearance and antioxidant defense [67, 68]. The elevation of its downstream metabolites (gulonate and threonic acid) in our study implies accelerated ascorbate degradation under oxidative stress, consistent with previous reports of L-diketogulonate accumulation in human erythrocytes during redox imbalance [69]. Erythronic acid, derived from erythritol catabolism via PPP [70], is linked to heightened metabolic flux, as corroborated by upstream PPP intermediates (ribulose and xylulose) in our MDD group. In the dysregulated PPP-associated antioxidant system, the imbalance in NADPH secretion exacerbates oxidative stress. These perturbations coalesce within the pentose-glucuronate metabolic network, where glucuronate pathway activation may further drive ascorbate degradation product accumulation, which suggests a potential metabolic reprogramming induced by redox imbalance, is associated with biochemical alterations in depression (Fig. 5).

Recent studies have reported that erythritol/erythronic acid may serve as oxidative stress biomarkers [71], positively correlated with hyperglycemia, hypertension, higher BMI, and central obesity in non-obese healthy individuals [72, 73]. In contrast, we observed divergent correlation patterns in MDD: both metabolites showed inverse associations with BMI (threonic acid: *r* = -0.062; erythronic acid: *r* = -0.069, Fig. 4), aligning with the negative BMI-depression relationship (*r* = -0.064; Figure S4). This disease-driven reversal may suggest that chronic oxidative stress in depression may alter metabolic priorities, these metabolites toward stress-responsive pathways rather than their homeostatic metabolic functions. Future experiments are required to confirm these findings.

Our study has several limitations. First, the relatively small sample size, particularly the limited number of healthy individuals with low psychological resilience, resulted in imbalanced group comparisons. However, this exploratory study provides preliminary findings warrant further investigation into this population. Second, the cross-sectional design restricts the causal interpretation of the results. Given emerging evidence suggesting psychological resilience is a dynamic trait rather than a fixed characteristic [74], future studies should incorporate prospective longitudinal assessments to elucidate causal relationships between resilience and depression. Additionally, targeted metabolomics in plasma samples may have constrained the discovery of metabolites; future work could employ non-targeted metabolomics and validate findings in cerebrospinal fluid or other biospecimens.

## Conclusion

The MDD×resilience interaction was not significant, whereas the main effect of MDD was robust, demonstrating that disease status exerts a more pronounced influence on metabolic alterations in depression than psychological resilience. Within the high resilience stratum, we identified convergent disruptions in amino acid metabolism and pentose–glucuronate interconversion, driven by decreases in arginine, methionine and kynurenine and increases in threonic and erythronic acids. Notably, threonic and erythronic acids are products linked to carbohydrate/PPP-related antioxidant processing, and their elevation may reflect an oxidative-stress burden. A five-metabolite panel distinguished MDD from healthy controls (AUC = 0.811) and showed symptom-aligned correlations. Together, these signatures may serve as potential diagnostic biomarkers and indicators of biological vulnerability in ostensibly resilient individuals, and they warrant mechanistic studies to validate the pathophysiological hypotheses.

## Supplementary Information

Below is the link to the electronic supplementary material.

Supplementary Material 1

## Data Availability

The datasets used and/or analysed during the current study are available from the corresponding author on reasonable request.

## Abbreviations

MDD: Major Depressive Disorder
UPLC-MS/MS: Ultra performance liquid chromatography–tandem mass spectrometry
ERS: Ego Resiliency Scale
OPLS-DA: Orthogonal partial least squares discriminant analysis
AUC: Area under the curve
DSM-5: Diagnostic and Statistical Manual of Mental Disorders, 5th Edition
M.I.N.I: Mini International Neuropsychiatric Interview
HAMD-17: Hamilton Depression Rating Scale – 17 items
HAMA-14: Hamilton Rating Scale for Anxiety
HC: Healthy controls
HR: High resilience
LR: Low resilience
HRD: High resilience MDD patients
LRD: Low resilience MDD patients
HRC: High resilience healthy controls
LRC: Low resilience healthy controls
CTQSF: Childhood Trauma Questionnaire – Short Form
BMI: Body mass index
GVIF: Generalized variance inflation factor
FDR: False discovery rate
VIP: Variable importance in projection
KEGG: Kyoto Encyclopedia of Genes and Genomes
ROC: Receiver operating characteristic
SD: Standard deviation
TRP: Tryptophan
KYN: Kynurenine
SAMe: S-adenosylmethionine
PPP: Pentose phosphate pathway
ROS: Reactive oxygen species
GSH: Glutathione
