# Supplementary material for: Metabolic profiling in major depressive disorder with high psychological resilience: changes in amino acid and carbohydrate metabolism
Source: BMC Psychiatry. 2026 Jan 17;26:151. doi: 10.1186/s12888-026-07798-4 (PMC12895811; doi:10.1186/s12888-026-07798-4)
Supplement: Supplementary file 1 — Supplementary Material 1 [file 12888_2026_7798_MOESM1_ESM.docx]

**Supplementary Information**

This text file includes:

Supplemental tables and figures.

**Table S1. Interaction effect analysis between psychological resilience and MDD status on metabolite levels adjusted for age and gender**

| **Metabolite** | **logFC** | **Average Expression** | **t** | | **P** | **P.adj** | **B-statistic** |
| --- | --- | --- | --- | --- | --- | --- | --- |
| \| Methylglutaric.acid \| \| --- \| | 0.122 | 0.213 | 3.677 | ＜0.001 | | 0.053 | 0.192 |
| X2.2Dimethylsuccinic.acid | 0.114 | 0.216 | 3.1931 | 0.002 | | 0.147 | -1.236 |
| Glutamine | -97.704 | 610.204 | -3.066 | 0.002 | | 0.149 | -1.581 |
| Butyrylcarnitine | -0.124 | 0.267 | -2.624 | 0.009 | | 0.340 | -2.677 |
| Glycine | -50.534 | 253.767 | -2.590 | 0.010 | | 0.340 | -2.753 |
| Propionic.acid | 3.914 | 21.210 | 2.562 | 0.011 | | 0.340 | -2.817 |
| Palmitoylcarnitine | -0.068 | 0.283 | -2.431 | 0.0157 | | 0.418 | -3.104 |
| Homoserine | -1.606 | 7.093 | -2.280 | 0.023 | | 0.430 | -3.418 |
| Isovaleric.acid | 1.584 | 6.402 | 2.277 | 0.024 | | 0.430 | -3.425 |
| N.Acetylaspartic.acid | -0.066 | 0.591 | 2.250 | 0.025 | | 0.430 | -3.477 |

Linear models were fitted for each metabolite using the limma package in R, including psychological resilience status, MDD diagnosis, and their interaction term, with age and gender as covariates. Multiple testing correction was applied using the false discovery rate (FDR). The table lists unadjusted and FDR-adjusted P-values for the interaction terms. Only the top 10 metabolites with the lowest unadjusted P-values are shown. The table includes the log2 fold change (logFC), average expression, t-statistic (t), raw p-values (P), adjusted p-values (P. adj), and the log-odds of differential expression (B-statistic).

**Table S2. ANOVA results for BMI differences across resilience and MDD groups**

| **Factor** | **Degrees of Freedom** | **Sum of Squares** | **Mean Square** | **F** | **P** |
| --- | --- | --- | --- | --- | --- |
| MDD status | 1 | 60.430 | 60.430 | 2.680 | 0.103 |
| Psychological resilience | 1 | 3.320 | 3.320 | 0.147 | 0.702 |
| MDD status* Psychological resilience | 1 | 102.620 | 102.620 | 4.507 | 0.035 |

This table presents the analysis of variance (ANOVA) comparing the BMI differences between the MDD and resilience groups. The results show that there were no significant differences in BMI between the groups (P = 0.103), and psychological resilience did not significantly affect BMI (P = 0.702).

**Table S3. Significant differential metabolites showing main effects of MDD status adjusted for age and gender**

| **Metabolite** | **logFC** | **P** | **P.adj** | **Direction** |
| --- | --- | --- | --- | --- |
| Ornithine | 15.416 | ＜0.001 | 0.015 | ↑ |
| Arginine | -14.798 | ＜0.001 | 0.002 | ↓ |
| Citric acid | -10.065 | 0.003 | 0.031 | ↓ |
| Pyroglutamic acid | 9.290 | 0.003 | 0.031 | ↑ |
| Glutamic acid | 7.014 | 0.002 | 0.025 | ↑ |
| Palmitoleic acid | 4.950 | 0.006 | 0.048 | ↑ |
| Hydroxypropionic acid | -4.857 | 0.004 | 0.046 | ↓ |
| Fructose | -4.039 | 0.002 | 0.026 | ↓ |
| Methionine | -2.478 | 0.006 | 0.048 | ↓ |
| Arachidonic acid | 1.345 | 0.001 | 0.013 | ↑ |
| GCDCA | -1.053 | 0.005 | 0.048 | ↓ |
| Threonic acid | 1.011 | 0.000 | 0.001 | ↑ |
| Erythronic acid | 0.957 | 0.000 | 0.001 | ↑ |
| Kynurenine | -0.550 | 0.002 | 0.027 | ↓ |
| Propionylcarnitine | -0.201 | 0.002 | 0.027 | ↓ |
| Ribulose | 0.187 | 0.000 | 0.001 | ↑ |
| Phenylpyruvic acid | -0.133 | 0.000 | 0.000 | ↓ |
| TCDCA | -0.126 | 0.002 | 0.025 | ↓ |
| N Acetylneuraminic acid | 0.123 | 0.000 | 0.001 | ↑ |
| Xylulose | 0.113 | 0.002 | 0.028 | ↑ |

Metabolites showing significant main effects of MDD status after adjusting for age and gender. Metabolites are sorted by |logFC|. Only metabolites with adjusted p < 0.05 and |logFC| > 0.1 are presented. ↑: Positive logFC indicates upregulation in MDD patients compared to healthy controls; ↓: negative logFC indicates downregulation.

**Table S4. Differential metabolites identified between MDD patients and healthy controls in the low resilience subgroup**

| **Metabolite** | **LogFC** | **Average Expression** | **t** | **P** | **P.adj** | **B-statistic** |
| --- | --- | --- | --- | --- | --- | --- |
| Quinic.acid | -0.253 | 0.230 | -3.479 | ＜0.001 | 0.121 | -0.599 |
| Ornithine | 26.217 | 78.038 | 3.264 | 0.001 | 0.121 | -1.168 |
| Methylglutaric.acid | -0.114 | 0.223 | -3.150 | 0.002 | 0.121 | -1.458 |
| Phenylpyruvic.acid | -0.170 | 0.713 | -2.975 | 0.004 | 0.121 | -1.887 |
| X2.2.Dimethylsuccinic.acid | -0.115 | 0.229 | -2.903 | 0.004 | 0.121 | -2.058 |
| N.Acetylneuraminic.acid | 0.194 | 0.774 | 2.886 | 0.005 | 0.121 | -2.098 |
| Xylose | -1.770 | 5.344 | -2.862 | 0.005 | 0.121 | -2.154 |
| Glutamine | 76.795 | 602.935 | 2.838 | 0.005 | 0.121 | -2.208 |
| N.Acetylaspartic.acid | 0.0691 | 0.594 | 2.784 | 0.006 | 0.121 | -2.332 |
| Glycine | 44.643 | 252.676 | 2.768 | 0.007 | 0.121 | -2.369 |

This table summarizes the differential metabolite analysis comparing patients with MDD and healthy controls within the low resilience subgroup. The analysis was conducted using the limma package. Only the top 10 metabolites with the lowest unadjusted P-values are shown.


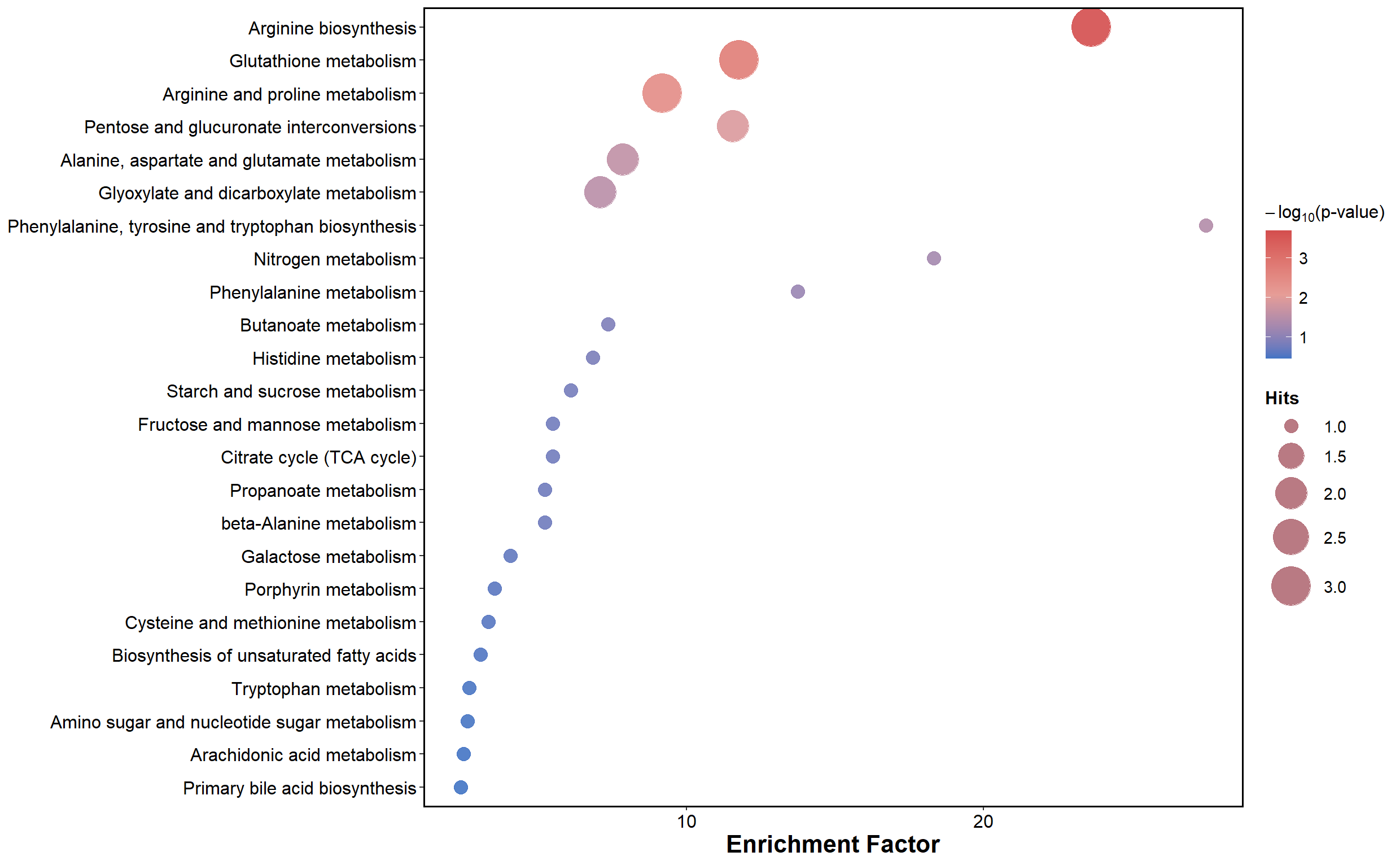


**Figure S1. KEGG pathway enrichment bubble plot of MDD main effects.** The x-axis represents the enrichment factor, the size of the bubble is proportional to the number of hits (mapped metabolites), and the color from blue to red reflects the significance level.


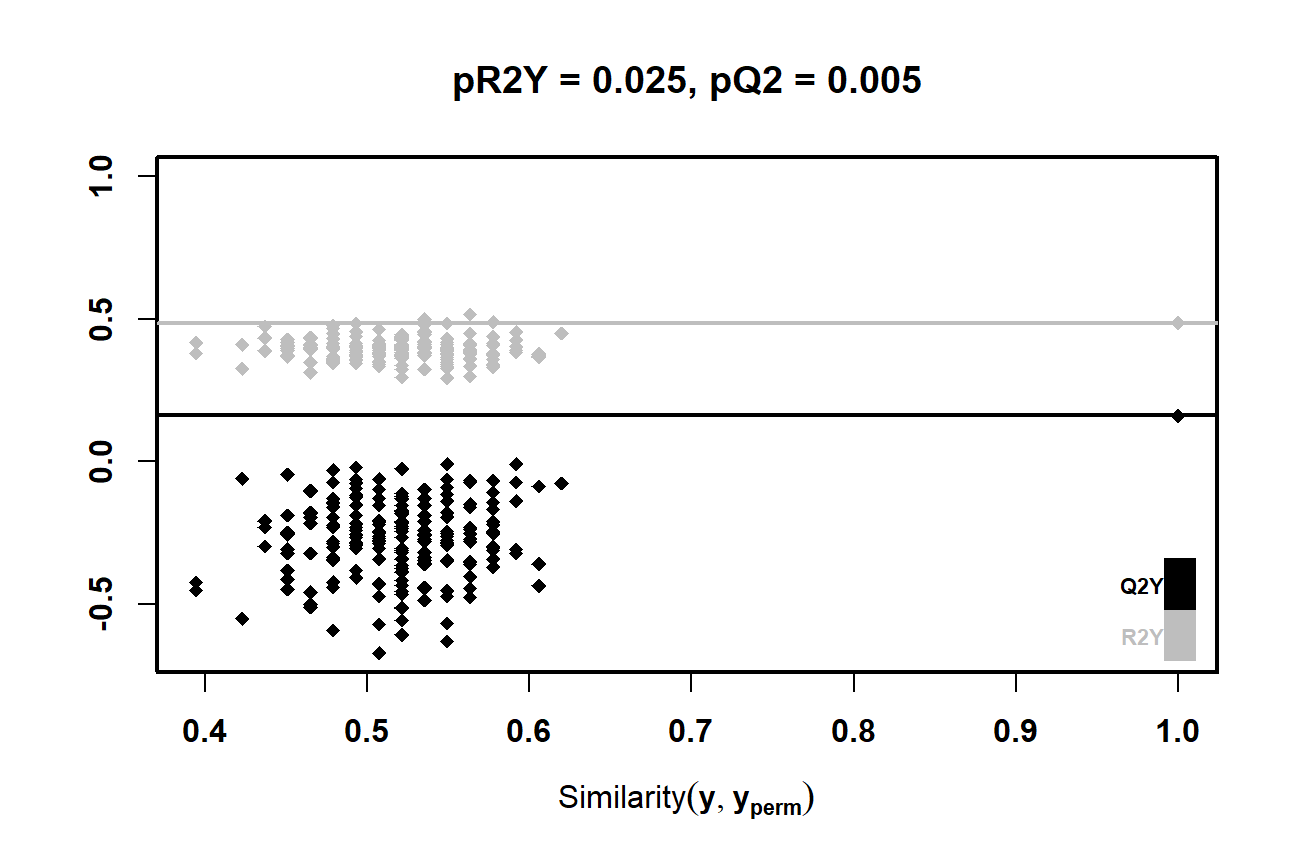


**Figure S2. 200-times permutation test of the OPLS-DA model.** The low pR2Y (0.025) and pQ2 (0.005) values indicate the model’s reliability and predictability without overfitting.


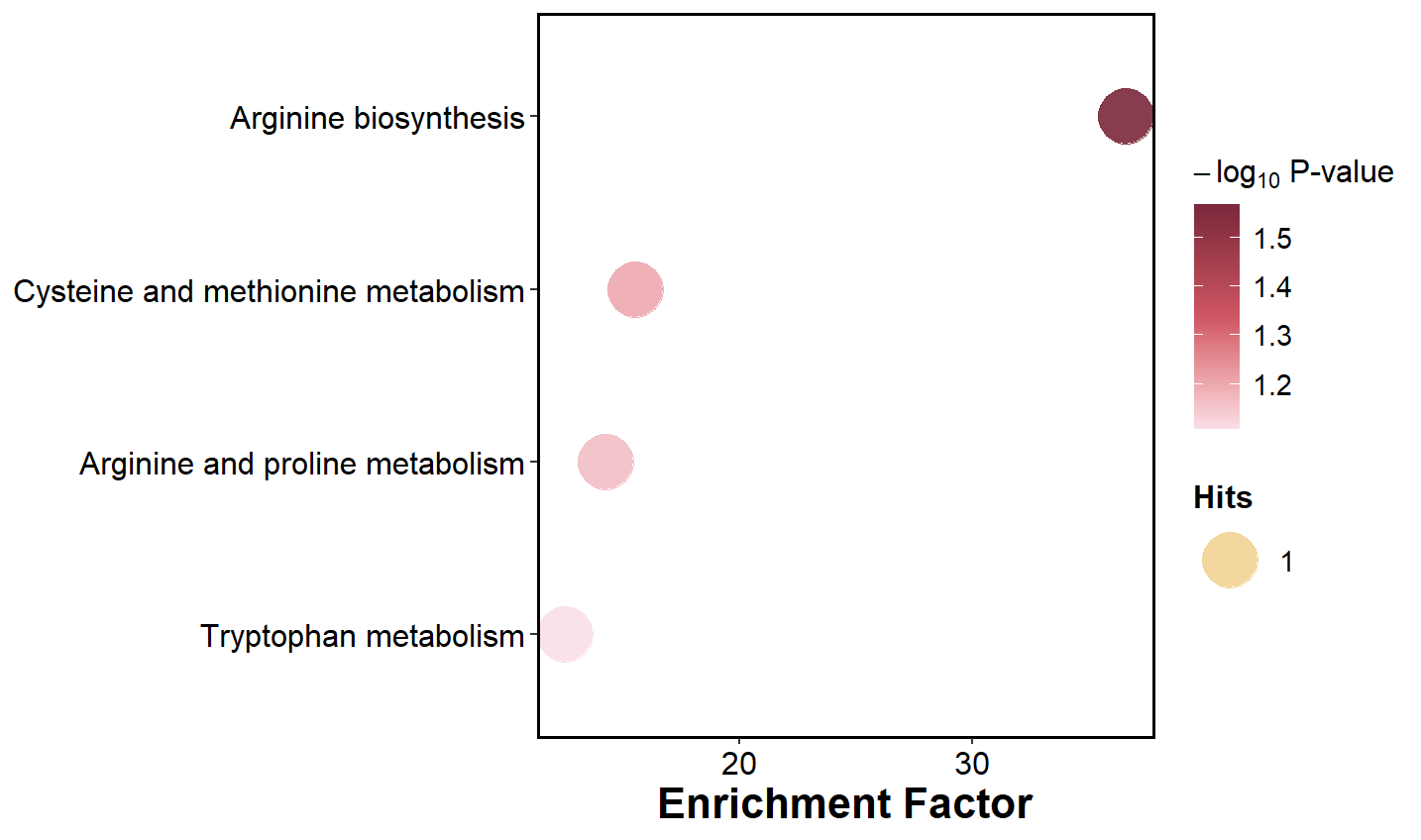


**Figure S3. KEGG pathway enrichment bubble plot of key differential metabolites.** The x-axis represents the enrichment factor, the size of the bubble is proportional to the number of hits (mapped metabolites), and the color from pink to deep red reflects the significance level.


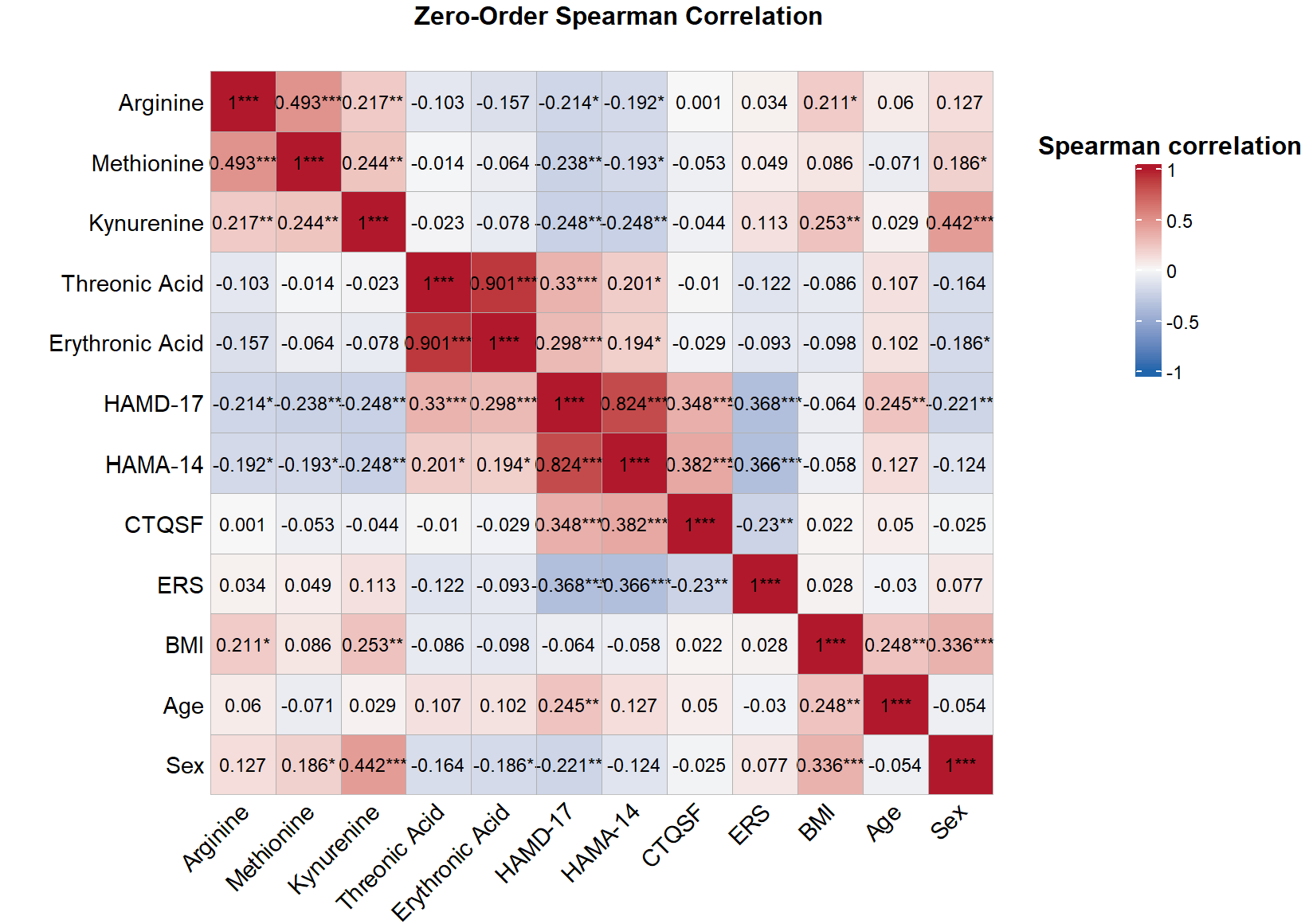


**Figure S4. Correlation of key metabolites, clinical scores, and demographic variables.** Positive correlations are shown in red, and negative correlations are shown in blue, with color intensity representing the correlation strength. Significant correlations are marked as follows: *P < 0.05, **P < 0.01, ***P < 0.001 (FDR corrected).
